# Thermal Conductivity of GaAs Nanowire Arrays Measured by the 3ω Method

**DOI:** 10.3390/nano12081288

**Published:** 2022-04-10

**Authors:** Ara Ghukasyan, Pedro Oliveira, Nebile Isik Goktas, Ray LaPierre

**Affiliations:** Department of Engineering Physics, McMaster University, Hamilton, Ontario, ON L8S 4L8, Canada; ghukasa@mcmaster.ca (A.G.); oliveirp@mcmaster.ca (P.O.); isikgokn@mcmaster.ca (N.I.G.)

**Keywords:** nanowire, twinning superlattice, thermal conductivity, thermoelectric

## Abstract

Vertical nanowire (NW) arrays are the basis for a variety of nanoscale devices. Understanding heat transport in these devices is an important concern, especially for prospective thermoelectric applications. To facilitate thermal conductivity measurements on as-grown NW arrays, a common NW-composite device architecture was adapted for use with the 3ω method. We describe the application of this technique to obtain thermal conductivity measurements on two GaAs NW arrays featuring ~130 nm diameter NWs with a twinning superlattice (TSL) and a polytypic (zincblende/wurtzite) crystal structure, respectively. Our results indicate NW thermal conductivities of 5.2 ± 1.0 W/m-K and 8.4 ± 1.6 W/m-K in the two samples, respectively, showing a significant reduction in the former, which is the first such measurements on TSL NWs. Nearly an order of magnitude difference from the bulk thermal conductivity (~50 W/m-K) is observed for the TSL NW sample, one of the lowest values measured to date for GaAs NWs.

## 1. Introduction

Interest in semiconductor nanowires (NWs) is motivated by finite size and surface effects that dominate the mechanisms of charge and phonon transport. NWs are particularly attractive as thermoelectric materials, [1,2] where efficiency is related to the dimensionless figure of merit [3].

(1) $$ZT=\frac{S^{2}\sigma T}{\kappa }$$

Here, T is the absolute temperature, S is the Seebeck coefficient (typically a few mV/K), σ is electrical conductivity , and κ is the total thermal conductivity:

(2) $$\kappa =\kappa_{e}+\kappa_{L}$$

The electronic component, $\kappa_{e}$, is proportional to σ and is often much smaller than the lattice component, $\kappa_{L}$, in semiconductors [4].

NWs stand to benefit thermoelectrics because an enhancement of the power factor ($S^{2}\sigma$) could be achieved in low-dimensional systems, as predicted by well-known theoretical studies [5,6]; however, these confined-carrier effects have not contributed significantly to thermoelectric improvements in NWs to date [7,8,9]. Another benefit is the lower thermal conductivity of NWs compared to the bulk, which has proven more practical for increasing ZT [10]. Among the III–V compounds, measurements on InAs [11,12,13,14] and GaAs [15] NWs have shown 30–80% reductions in thermal conductivity compared to their bulk value. The influence of structural and compositional features has also been demonstrated. For example, measurements on Si [7] and Si_0.96_Ge_0.04_ [16] NWs have revealed that surface roughness can grant an additional 70% reduction compared to smooth NWs. Core-shell GaAs-AlAs NWs measured by Juntunen et al. [17] showed a non-monotonic dependence on the shell thickness, and a minimal thermal conductivity near 1 W/m-K (versus ~50 W/m-K of bulk GaAs [18,19]). Li et al. [20] measured the thermal conductivity of Si/Si_0.9_Ge_0.1_ superlattice NWs, determining values of 5 to 6 W/m-K at room temperature, much lower than Si NWs of a similar size [21].

In addition to reduced thermal conductivity, good electrical conductivity (as well as a large Seebeck coefficient) is still required for a large ZT. Interfacial and surface roughness in NWs can accomplish the former, as numerous studies have demonstrated, but the need persists for more phonon-specific mechanisms to avoid additional electron scattering. As discussed below, NWs with sharp and periodic crystallographic interfaces [22], such as twinning superlattices (TSLs), could be ideal for this purpose. Emerging growth techniques are recently enabling the controlled synthesis of TSL NWs in patterned arrays via vapor–liquid–solid (VLS) methods [23].

**Figure 1:**
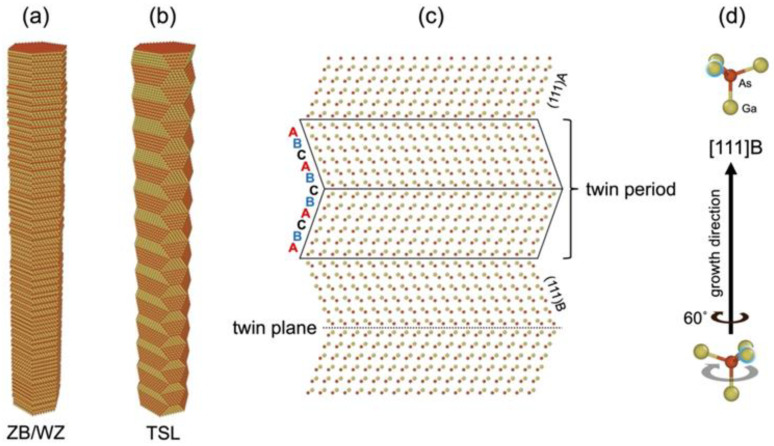
Three-dimensional structures for (**a**) polytypic zincblende/wurtzite (ZB/WZ) and (**b**) twinning superlattice (TSL) nanowires, showing [111]A and [111]B surface faceting in the latter. (**c**) Bilayer stacking in the TSL, seen in an orthogonal projection along $[11\bar{2}$], exhibiting reversal of the normal ABC stacking sequence across the twin plane. (**d**) Twinning is equivalent to a rotation of the crystal structure about the NW axis by 60°, illustrated by tetrahedral primitives of bulk GaAs. The indicated growth direction applies to the entire figure.

In a handful of III–V compounds including GaAs [24], both the zincblende (ZB) and wurtzite (WZ) phases are accessible during growth and can be selected by adjusting the growth conditions [25,26]. Polytypic (ZB/WZ) [27], phase-modulated [28], and twinning-ZB [24,26,29,30] III–V NWs can be produced in this way. Among these, disordered NW structures (Figure 1a) are associated with low thermal conductivity [27] but also low electron mobility [31], which is undesirable for devices. More ordered structures are also possible, such as twinning superlattices (TSLs; Figure 1b–d), where complementary twin segments form repeating sections that periodically rotate by 60° about the NW axis. For thermoelectric device applications, TSLs may provide a means of coherent phonon engineering [32] based on adjusting the twin period for minimal lattice thermal conductivity, as suggested by computational studies [33,34,35] and existing work on heterojunction superlattices [36]. Due to the novelty of TSL NWs and corresponding challenges in their synthesis, experimental measurements of the thermal conductivity are absent from the literature. This work provides, to the best of our knowledge, the first of such measurements on TSL NWs within the III–V material system and perhaps on TSL NWs in general.

Many of the NW thermoelectric devices proposed to date [37,38,39,40,41,42,43] employ a composite architecture, with an interstitial material introduced for mechanical support and planarization of the NW array. Among the thermal conductivity measurements reported above, the thermoreflectance-based approaches of Persson et al. [13] and Juntunen et al. [17] are applied directly to measurement devices of this type. In this case, heat flow remains highly one-dimensional, so a linear effective-medium model can be used to extract the NW thermal conductivity [13].

As an alternative to thermoreflectance measurements, we employ the AC 3ω method [44,45], which has been used previously to measure the thermal conductivity of NWs embedded in nanoporous Al_2_O_3_ [46,47,48]. In comparison to embedded (electrodeposited) NWs, free-standing VLS-grown NWs offer greater flexibility in controlling the composition and crystal structure, as mentioned earlier, with the added benefit of direct integration on Si substrates. Using a spin-on polymer for planarization, as in Refs. [13,17] and various other NW devices [49], allows for the 3ω method to be applied as a generic means of measuring the array thermal conductivity. Compared to traditional techniques, the 3ω method requires far shorter equilibration times and is insensitive to radiative losses [44]. Compared to optical techniques [13,17], the thermal penetration depth [45] is much greater in the 3ω method, because typical measurement frequencies are on the order of kHz (as opposed to MHz).

To examine the influence of crystal structure on the NW thermal conductivity, and to illustrate a novel adaptation of the 3ω method, we report measurements on GaAs NW arrays featuring polytypic ZB/WZ NWs (Figure 1a) versus twinning superlattice (TSL) NWs (Figure 1b–d). In the sections that follow, we describe the VLS growth of these NWs in dense and free-standing arrays, followed by a characterization of the crystal structure, and fabrication of the NW-composite measurement device. Next, an overview of the 3ω method is provided, with a discussion of theoretical models and the practical details of implementation. Finally, results are discussed in context with available experimental and theoretical data. Abbreviations and symbols are tabulated in Table A6 of Appendix D.

## 2. Materials and Methods

### 2.1. Nanowire Growth and Characterization

Arrays of GaAs NWs were grown on 300 μm thick p^+^-Si substrates (ρ≤0.005 Ω-cm) with a <111> surface orientation. GaAs NWs were grown in a 2 × 2 mm^2^ area on the substrate surface by the self-assisted (SA) VLS method with a Ga droplet as the seed particle, using gas source molecular beam epitaxy. NW growth details are provided in Appendix A. Two samples were grown with polytypic ZB/WZ NWs (sample A) and twinning superlattice (TSL) NWs (sample B) using identical processes, apart from a Be dopant flux introduced in the latter that induces a TSL structure due to changes in the NW sidewall surface energy [26].

**Figure 2:**
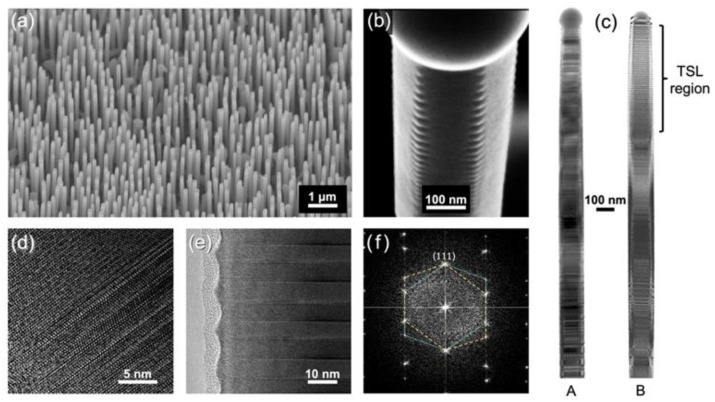
(**a**) SEM image of the NW array from sample B; (**b**) SEM image of a NW top, showing surface faceting due to a TSL; (**c**) Side-by-side TEM images of a polytypic NW from sample A (left) and a TSL NW from sample B (right); (**d**) HRTEM image near the center of the same NW from sample A, showing a polytypic WZ/ZB NW structure; (**e**) HRTEM image near the center of the TSL in sample B; (**f**) Selective-area electron diffraction pattern confirming ZB twins in the TSL region of sample B.

After growth, the NW arrays were characterized by scanning electron microscopy (SEM), bright-field transmission electron microscopy (TEM), and high-resolution TEM (HRTEM). Samples were prepared for TEM by mechanically transferring the NWs to a Cu grid. TEM was performed using a *JEOL 2010F* with 200 kV accelerating voltage. Selective-area electron diffraction (SAED) was performed in the TEM to confirm crystal structure. SEM was performed using a *JEOL 7000F*. SEM confirmed a dense and uniform NW array (Figure 2a) from sample B with similar results to sample A with comparable NW length and diameter.

The growth procedure produced a TSL structure near the top of the NW in sample B, observed by the surface faceting in SEM (Figure 2b). A side-by-side comparison, shown in Figure 2c, highlights the structural differences between samples A and B. The crystal structure of sample A is ZB twins with WZ insertions (denoted ZB/WZ, as depicted in Figure 1a) as confirmed by HRTEM in Figure 2d. The crystal structure of sample B was identical to sample A, except near the top third of the NW where the polytypic structure was replaced with a TSL structure (depicted in Figure 1b,c), confirmed by HRTEM in Figure 2e and SAED in Figure 2f. The Ga droplet that seeded the NW growth is observable at the top of the NW in Figure 2b,c.

### 2.2. Device Fabrication

**Figure 3:**
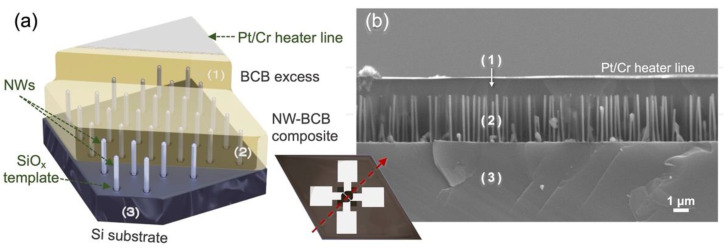
Diagrams illustrating the device layers used to model heat conduction: (1) the electrically insulating BCB excess, (2) the target NW-BCB composite layer, and (3) the silicon substrate. (**a**) 3D schematic of the device, with an inset showing the heater line and four contact pads. (**b**) Cross-sectional SEM image of a device from sample B.

The 3ω measurement device is depicted in Figure 3. To fabricate the device, layers of benzocyclobutene (BCB) were applied to the NW arrays by repeated spin-coating at 7000 rpm for 77 s, followed by a 1-h cure at 250 °C in an inert N_2_ atmosphere after each coat. In this way, the NWs were completely submerged and insulated by an additional 3.5 μm of BCB above. A layer of *microposit S1827* photoresist was then spin-coated on top of the cured BCB at 3500 rpm for 30 s, and a 1 mm^2^ opening was developed in the photoresist, above a suitable area of the NW array. Using the photoresist as an etch mask, a 0.5 μm deep cavity was etched into the excess BCB using reactive ion etching under 50 W power, with 35.8 sccm CF_4_, 5.4 sccm O_2_, and 1.8 sccm N_2_. This step removed some of the BCB above the measurement section of the NW array while allowing shorts to be avoided between the heater and longer parasitic NWs. The etch mask was then dissolved in an acetone bath.

The height of the remaining BCB excess was later measured by cross-sectional SEM, as seen in Figure 3b. In the final fabrication step, Cr (70 Å) and Pt (1500 Å) metal were deposited on the BCB by evaporation through a mask bearing the heater pattern, and the heater line was positioned diagonally across the cavity etched above the NW array. After deposition, another acetone bath was used to remove the resist and excess metal by lift-off, forming the 30 × 1000 μm heater line and four contact pads illustrated in the inset of Figure 3a.

### 2.3. 3ω Measurements

A *Stanford Research Systems SR810* digital lock-in amplifier was used for AC measurements across a range of frequencies. The complex temperature rise (${\tilde{\theta }}_{2\omega ,\mathrm{rms}}$) of the heater line was then calculated from the relation [50]:

(3) $${\tilde{\theta }}_{2\omega ,\mathrm{rms}}={\left[\frac{I_{1\omega ,\mathrm{rms}}}{\sqrt{2}}\times \frac{dR}{dT}\right]}^{-1}\left(V_{3\omega ,\mathrm{rms},\mathrm{re}}-iV_{3\omega ,\mathrm{rms},\mathrm{im}}\right)$$

where $I_{1\omega ,\mathrm{rms}}$ is the sinusoidal current through the heater line at the source frequency, and $V_{3\omega ,\mathrm{rms},\mathrm{re}}$ and $V_{3\omega ,\mathrm{rms},\mathrm{im}}$ are the in- and out-of-phase components of the third harmonic voltage across the heater line. In Equation (3), the resistance coefficient (dR/dT) enables the coupling of voltage and temperature via the temperature dependence of the line resistance. Resistance coefficients were obtained using a *Thorlabs TED4015* temperature controller together with a Peltier element to sweep sample temperature from 15 to 30 °C, while a *Keithley 2000* series multimeter was used to measure line resistances (see Figures S1–S3 in the Supplementary File, Section I). All AC measurements were done with the sample in a vacuum. The heater was contacted by copper probes in a typical four-point configuration with the substrate pressed against an aluminum block. Prior to the 3ω measurement, the electrical resistance was measured between the heater line and the aluminum block (i.e., ground) to confirm the lack of a leakage current through the sample. These measurements yielded resistances exceeding 40 MΩ, owing to the highly insulating BCB. (A small resistance would indicate significant power dissipation inside the sample, invalidating the assumed heat flow model [45].) In addition to samples A and B, a third BCB-on-Si sample (sample C) was measured to obtain the baseline thermal conductivity of the BCB. A 400 μm thick Si substrate (ρ>1000 Ω-cm) was used for sample C.

The standard 3ω measurement circuit (as in Figure 2b in Ref. [51]) was used to obtain the $V_{3\omega ,\mathrm{rms}}$ components and the current $I_{1\omega ,\mathrm{rms}}=V_{1\omega ,\mathrm{rms}}/R_{\mathrm{ref}}$, which was produced by a constant-current source and measured via the voltage across a precision resistor ($R_{\mathrm{ref}}$). Using a single lock-in amplifier, the 1ω current measurements were conducted immediately after the 3ω voltage measurements, over the same set of frequency points. Connections were switched externally (at the lock-in amplifier) and the contacted sample was not disturbed in any way. All measurements were conducted using the digital interface of the lock-in amplifier. We took the average reading over 10 s of continuous measurement at each frequency. Uncertainty was estimated from the standard deviation of these values, then propagated to ${\tilde{\theta }}_{2\omega ,\mathrm{rms}}$ via Equation (3), whereby the error on dR/dT (less than 2%; see Supplementary File, Section I) was also incorporated.

### 2.4. 3ω Data Fitting

To fit the measured temperature data (from Equation (3)), the two-dimensional heat equation can be solved analytically, assuming a uniform heat flux between the line heater and the top layer [45,51]. This gives the complex temperature rise of the heater as a function of the angular frequency, ω:

(4) $${\tilde{T}}_{h}\left(\omega \right)=\frac{\Delta \tilde{T}+pr_{\mathrm{th}}}{1+i2\omega C_{h}d_{h}\left(\frac{\Delta \tilde{T}+pr_{\mathrm{th}}}{p}\right)}$$

where

(5) $$\Delta \tilde{T}\left(\omega \right)=-\frac{P}{\pi l\kappa_{⊥,1}}\int_{0}^{\infty }\frac{1}{A_{1}B_{1}}\frac{\sin^{2}\left(b\lambda \right)}{b^{2}\lambda^{2}}\;d\lambda$$

Here, P is the peak electrical power, while p=P/2bl is the heat flux, b is half the width of the heater line, and l is the length of the heater line. The integration variable, λ, is an inverse length. The parameters $C_{h}$, $d_{h}$, and $r_{\mathrm{th}}$ represent the volumetric heat capacity, thickness, and thermal contact resistance, respectively, of the heater. In Equation (5), $\kappa_{⊥,1}$ is the cross-plane thermal conductivity of the top layer (i.e., the insulating BCB). The remaining thermophysical properties are contained in the coefficients $A_{1}$ and $B_{1}$, which are defined by

(6) $$A_{n-1}=\frac{A_{n}\frac{B_{n}\kappa_{⊥,n}}{B_{n-1}\kappa_{⊥,n-1}}-\tanh\left(B_{n-1}d_{n-1}\right)}{1-A_{n}\frac{B_{n}\kappa_{⊥,n}}{B_{n-1}\kappa_{⊥,n-1}}\tanh\left(B_{n-1}d_{n-1}\right)}\;$$

and

(7) $$B_{n}=\sqrt{\psi_{n}\lambda^{2}+\frac{i2\omega C_{n}}{\kappa_{⊥,n}}}\;$$

for layer indices n=2, …, N, numbered in increasing order from the second layer down to the substrate (layer N), as in Figure 3. The quantity $\psi_{n}$ is the anisotropy ratio, defined as $\psi_{n}=\kappa_{∥,n}/\kappa_{⊥,n}$. The coefficient $A_{1}$ of the uppermost layer is determined recursively from Equation (6) and the recurrence is terminated at the substrate layer, where $A_{N}\equiv -1$. The coefficients $B_{n}$, on the other hand, are calculated directly form Equation (7). In this way, the base temperature rise in Equation (5) considers the accumulated influence of layers in the sample, while Equation (4) includes a correction accounting for the physical heater line [45]. Thermophysical properties of the sample are extracted by fitting this model (Equations (4) and (5)) to the measured temperature data (Equation (3)).

The data were fitted by minimizing the mean-square-error (MSE) defined by

(8) $$\bar{\epsilon }\left(\vec{\chi }\right)=\;\frac{1}{M}\sum_{k=1}^{M}\|{\tilde{\theta }}_{2\omega_{k}}-{\tilde{T}}_{h}{\left(\omega_{k},\;\vec{\chi })\right\|}^{2}$$

where the vector $\vec{\chi }$ contains the fitting parameters. Each layer in a sample admits four individual parameters, namely the cross-plane thermal conductivity $\kappa_{⊥,n}$, the volumetric heat capacity $C_{n}$, the layer thickness $d_{n}$, and the anisotropy ratio $\psi_{n}$.

Samples A and B were modelled as three-layer structures (N=3), consisting of (1) the insulating BCB in contact with the heater, (2) the NW-BCB composite, and (3) the Si substrate, as shown in Figure 3. Sample C was modelled as a two-layer structure (N=2), consisting only of (1) a uniform BCB layer and (2) a Si substrate.

The final thermal conductivity of the NW array ($\kappa_{\mathrm{NW}}$) was calculated from the total thermal conductivity of the NW-BCB composite layer using an effective-medium model [13]:

(9) $$\kappa_{\mathrm{NW}-\mathrm{BCB}}=x\kappa_{\mathrm{NW}}+\left(1-x\right)\kappa_{\mathrm{BCB}}\;$$

Here, the variable 0≤x≤1 represents the volume fraction of NWs in layer (2). The volume fraction must account for the NW growth yield because a fraction of oxide holes will not nucleate a NW due to parasitic effects. The yield was estimated by counting all NWs (roughly 4000 individuals) in a 30 × 30 μm^2^ area of each NW array versus the known density of nucleation sites. The measured thermal conductivities, $\kappa_{\mathrm{NW}-\mathrm{BCB}}$ and $\kappa_{\mathrm{BCB}}$, were obtained by fitting layer (2) of samples A or B and layer (1) of sample C, respectively.

## 3. Results and Discussion

**Figure 4:**
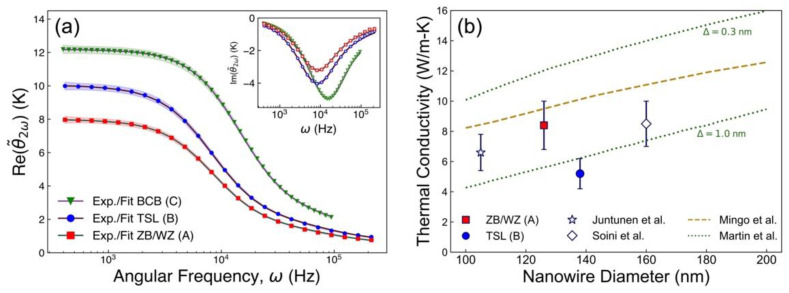
(**a**) Measured temperature amplitudes for samples A, B, and C, along with fitted curves, for the in-phase and out-of-phase (inset) components of each. (**b**) Thermal conductivity of GaAs NWs, with the polytypic ZB/WZ NWs (red, sample A) and the TSL NWs (blue, sample B) data points corresponding to measurements in panel (**a**). For comparison, the white markers indicate the experimental results of Juntunen et al. [17] and Soini et al. [15]. The dashed and dotted lines represent theoretical results from Mingo et al. [55] and Martin et al. [56], respectively, with Δ indicating surface roughness for the latter.

**Figure A1:**
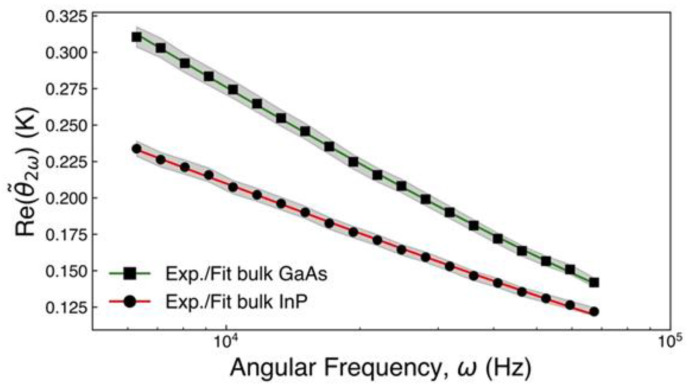
Measured and fitted temperature amplitudes for bulk GaAs and InP samples. Shaded regions around the data points indicate the measurement uncertainty.

The 3ω measurement data is shown in Figure 4a for samples A (red), B (blue), and C (green) along with curves corresponding to the fitted material properties. We considered three separate paradigms for the fit model vis a vis Equations (4) and (5); fitting with (i) the heater contribution neglected ($C_{h}d_{h}=0$, $r_{\mathrm{th}}=0$), (ii) the heater thermal mass neglected ($C_{h}d_{h}=0$, $r_{\mathrm{th}}\neq 0$), and (iii) all heater corrections included ($C_{h}d_{h}\neq 0$, $r_{\mathrm{th}}\neq 0$). Uncertainties on the fitted parameter values were estimated by taking the largest range within the measurement error, which is indicated by the shaded regions in Figure 4a. The best overall fit, in terms of the minimal MSE, was achieved using paradigm (ii). Paradigm (iii) produced comparable results for the NW and BCB thermal conductivities, agreeing with (ii) within uncertainty. As can be expected [45], the thermal mass of a $d_{h}=157$ nm heater line had only a small effect on the extrapolated values. Inclusion of the thermal resistance, on the other hand, greatly improved the fit. Figure S4 in Section II of the Supplementary File confirms the sensitivity of the heat model, Equations (4)–(7), to the thermal conductivities of the upper two layers vis a vis the finite penetration depth [45,51] of the temperature oscillations, shown in Figure S5, as compared to the thicknesses of layers (1) and (2) in our samples. The complete parameters are tabulated in Table A1, Table A2 and Table A3 in Appendix B. Measurements on bulk GaAs and InP substrates were used to validate the experimental setup, producing results in excellent agreement with the well-known bulk thermophysical properties of these materials [52,53,54], as shown in Figure A1 and Table A4 and Table A5 of Appendix C.

To consider thermal contact resistance between the sample layers, we used the slightly modified model of Olson et al. [57] to extend Equations (4)–(7). This approach effectively yielded zero resistance between layers (1)–(2) and between layers (2)–(3) in both samples A and B. A finite thermal resistance between the Cr/Pt heater line and the BCB of layer (1) was necessary to fit the high frequency data ($\omega >10^{4}$ Hz) shown in Figure 4a. $r_{\mathrm{th}}=0.02\pm 0.01$ cm^2^-K/W was obtained for both samples A and B.

The array thermal conductivities were determined from NW volume fractions $x_{A}=0.056\pm 0.006$ and $x_{B}=0.084\pm 0.009$ for samples A and B, respectively. Volume fractions were calculated from yields 0.50±0.05 and 0.63±0.05, and NW diameters $D_{A}=126\pm 5$ nm and $D_{B}=138\pm 5$ nm, for samples A and B, respectively. The thermal conductivity of BCB was 0.19±0.03 W/m-K obtained from sample C. The thermal conductivities for the disordered polytypic ZB/WZ NWs (sample A) and the TSL NWs (sample B) were $\kappa_{A}=8.4\pm 1.6$ W/m-K and $\kappa_{B}=5.2\pm 1.0$ W/m-K, as shown in Figure 4.

As a point of comparison, the bulk GaAs thermal conductivity of ~50 W/m-K [18,19] would give a thermal conductivity of ~3 W/m-K at the same volume fraction of samples A or B, where ~0.6 W/m-K was in fact measured for the NW-BCB layers. The NW thermal conductivities extracted from the samples are indeed much smaller than 50 W/m-K. The thermal conductivity of the ZB/WZ NWs from sample A was in the same range as prior theoretical and experimental results, as shown in Figure 4b (red data point). Here, the theoretical curves taken from Mingo et al. [55] (dashed) and Martin et al. [56] (dotted) indicate the approximate size dependence of the thermal conductivity for NWs with fully diffuse phonon-boundary scattering and for NWs with boundaries characterized by a root-mean-square roughness, Δ, respectively. Apart from roughness, the rate of phonon-boundary scattering is inversely proportional to the NW diameter and imparts most of the size dependence to the thermal conductivity of NWs at the diameters studied [55,56]. Based on these trends, the ZB/WZ NWs have a proportionally larger thermal conductivity compared to the 105 nm diameter NWs from Ref. [17]; however, the TSL NWs from sample B (blue data point in Figure 4b) exhibit a decrease in thermal conductivity within experimental uncertainty, despite even larger diameter. This result is among the lowest thermal conductivities achieved to date for GaAs NWs.

Lower values may yet be attainable by carefully adjusting the TSL period. As in traditional superlattices [36,58], there should exist an optimum twin period that minimizes the thermal conductivity of TSL NWs [33,34,35]. The mechanism of thermal conductivity reduction, while not fully understood [59], is usually attributed to both coherent and incoherent phonon transport in superlattices [60,61]. A fraction of phonon modes experience repeated reflections between interfaces, leading to resonances that inhibit axial propagation. Conversely, the same interfaces form resistive barriers for phonons that decohere at shorter length scales. In this view, the superlattice period selects the phonon modes (frequencies and wavelengths) affected by either scattering regime, resulting in a tunable thermal conductivity depending on the contribution of these modes to heat conduction in the periodic structure. Compared to traditional heterojunctions [20], the inherent sharpness of TSL interfaces in NWs should improve phonon coherence and provide greater tunability.

The TSL structure can be formed in NWs of various materials, provided compatible symmetry of the crystal lattice and energetic favorability for formation, as determined by growth conditions and atomic bonding. While GaAs NWs are easier to synthesize, TSLs have been observed in NWs of other III–V [22,29,30,32] and II–V compounds [62]. Because thermal conductivity is phenomenologically similar in these crystalline materials, similar behavior can be expected for TSLs. In particular, TSL NWs of materials with a lower bulk thermal conductivity than GaAs (for example, InAs and InSb [52]) merit future consideration.

## 4. Conclusions

In summary, we measured the thermal conductivity of GaAs NW arrays with polytypic ZB/WZ versus TSL structure, using a NW-BCB composite device structure that we adapted for the 3ω method. The thermal conductivity of GaAs TSL NWs was measured for the first time, and the result extracted from an effective-medium model indicated a thermal conductivity of $\kappa_{B}=5.2\pm 1.0$ W/m-K, versus $\kappa_{A}=8.4\pm 1.6$ W/m-K for the polytypic sample. A lower thermal conductivity was observed in the TSL sample, despite slightly larger diameter, marking a distinct reduction. Analysis of the 3ω data indicated a significant thermal contact resistance between the BCB polymer used for planarization and the Pt/Cr heater line used for thermometry. Thermal contact resistances between sample layers, however, were found to be negligible.

These results pave the way for future systematic studies aimed at understanding the effect of crystal structure on heat flow in III–V NWs and outline an electrical characterization method with a precision comparable to thermoreflectance-based approaches. The tenfold reduction from the bulk thermal conductivity observed in our TSL NWs represents an encouraging result of NW thermoelectrics. Further reductions in the thermal conductivity may be possible by tuning the period of the TSL structure (the twin segment length), varying the NW diameter, and inducing the TSL structure in other NW materials.

## Data Availability

Data is contained within the article and Supplementary Materials.

## Supplementary Materials

The following supporting information can be downloaded at: https://www.mdpi.com/article/10.3390/nano12081288/s1, Figures S1–S3: temperature dependence of the line resistances of sample A, B, and C; Figure S4: sensitivity of the heat model to various sample parameters; Figure S5: thermal penetration depth in BCB and NW-BCB layers.

## Appendix A

Using plasma-enhanced chemical vapor deposition, 30 nm of SiO_x_ was deposited on the substrate surface. To provide NW nucleation sites, arrays of holes were etched through the surface oxide in a hexagonal array pattern with a pitch of 360 nm, using electron beam lithography and reactive ion etching. The total extent of the array was confined to a 2 × 2 mm^2^ area on the substrate surface, as defined by the oxide template. GaAs NWs were grown by the self-assisted vapor–liquid–solid method, with a Ga droplet as the seed particle, using gas source molecular beam epitaxy. Ga was supplied from an effusion source and group V elements were supplied as dimers from a hydride gas cracker.

The polytypic zincblende/wurtzite NWs (sample A) and TSL NWs (sample B) arrays were grown using identical processes, apart from the dopant flux introduced in the latter that is used to induce a TSL structure [26]. A 250 s Ga pre-deposition and the brief growth of a short GaP base were employed to improve the NW yield [63]. GaAs NWs were then grown in three segments: (i) 50 min at a substrate temperature of 630 °C, (ii) a ramp down to 537 °C over the next 50 min, and (iii) a final 30 min of growth at 537 °C. This process ensured a high NW yield at the beginning of growth, followed by a low temperature required for TSL formation in sample B. During the growth of sample B, a Be dopant flux supplied from an elemental effusion cell was introduced in segment (i) at a NW dopant concentration of $6.0\times 10^{17}$ cm^−3^, then ramped up in segment (ii), and finally held at $3.0\times 10^{19}$ cm^−3^ in segment (iii). The Be dopant flux was increased gradually to avoid Ga droplet instability associated with a high dopant flux. The incorporated dopant concentration was based on previous calibrations [64,65] and no intentional dopants were introduced in sample A.

## Appendix B

The thermophysical properties of all layers across the three samples, as determined by three fitting paradigms, (i)–(iii), are tabulated in Table A1, Table A2 and Table A3. Error estimates are included on the parameter values for which the model exhibits high sensitivity (see the Supplementary File, Section II). The results for the anisotropy ratio ($\kappa_{∥}/\kappa_{⊥}$) were on the order of $10^{-7}$ for both sample A and sample B, suggesting highly one-dimensional heat flow through the NW-BCB composites. For the uniform BCB layer in sample C, we fixed $\psi_{1}=1$.

Reference value ranges are provided, where available, in the rightmost columns of each table. All entries in the columns (i)–(iii) represent fitted parameter values for the corresponding fit paradigm, except for underlined values, which indicate constant parameters, and values of the MSE, $\bar{\epsilon }$. Fitted parameters exceeding their reasonable physical range are emphasized in italic text.

The inclusion of $r_{\mathrm{th}}\neq 0$ improves the MSE by at least an order of magnitude, so paradigm (i) was discounted. Regarding paradigms (ii) and (iii), we note that thermal conductivity results for the BCB and NW-BCB layers agree within uncertainty for all three samples. Indeed, the heater thermal mass has only a small effect on the measured thermal conductivity (as per Ref. [45] (p. 2146)), considering the relative dimensions and heat capacity of the heater line versus that of the target layers. Paradigm (ii) was chosen over (iii) because it produced no unphysical values as well as the lowest overall MSE across the three samples.

**Table A1:** Fitted parameter values for sample A.

Parameter	Units	(i) $C_{h}d_{h},\;r_{th}=0$	(ii) $r_{th}\neq 0$	(iii) $C_{h}d_{h},r_{th}\neq 0$	Ref. Value
$\kappa_{\mathrm{BCB}}$	W/m-K	0.55 ± 0.03	0.19 ± 0.04	0.17 ± 0.04	0.18–0.29 [66,67,68,69]
$C_{\mathrm{BCB}}$	J/cm^3^-K	1.10 ± 0.03	1.88 ± 0.04	1.99 ± 0.04	2.19–2.29 [66,70]
$\kappa_{\mathrm{NW}-\mathrm{BCB}}$	W/m-K	0.20 ± 0.05	0.65 ± 0.04	0.70 ± 0.06	
$C_{\mathrm{NW}-\mathrm{BCB}}$	J/cm^3^-K	*3.70* × *10^−7^*	0.41	*4.57* × *10^−7^*	
$\kappa_{p^{+}\mathrm{Si}}$	W/m-K	266	67	46	35–55 [71]
$C_{p^{+}\mathrm{Si}}$	J/cm^3^-K	0.0920	1.22	0.97	-
$r_{\mathrm{th}}$	cm^2^-K/W	0	0.0114	0.028	-
$C_{h}$	J/cm^3^-K	0	0	2.82	2.82 [52]
$d_{h}$	nm	0	0	157	-
$\bar{\epsilon }$	10^−3^ K^2^	19.2	2.25	1.92	

**Table A2:** Fitted parameter values for sample B.

Parameter	Units	(i) $C_{h}d_{h},\;r_{th}=0$	(ii) $r_{th}\neq 0$	(iii) $C_{h}d_{h},r_{th}\neq 0$	Ref. Value
$\kappa_{\mathrm{BCB}}$	W/m-K	0.47 ± 0.02	0.19 ± 0.04	0.17 ± 0.04	0.18–0.29 [66,67,68,69]
$C_{\mathrm{BCB}}$	J/cm^3^-K	1.18 ± 0.04	1.93 ± 0.04	2.02 ± 0.04	2.19–2.29 [66,70]
$\kappa_{\mathrm{NW}-\mathrm{BCB}}$	W/m-K	0.20 ± 0.05	0.61 ± 0.04	0.68 ± 0.07	
$C_{\mathrm{NW}-\mathrm{BCB}}$	J/cm^3^-K	2.91	0.38	0.37	
$\kappa_{p^{+}\mathrm{Si}}$	W/m-K	*8.46* × *10^5^*	57	48	35–55 [71]
$C_{p^{+}\mathrm{Si}}$	J/cm^3^-K	4.68	2.02	2.05	-
$r_{\mathrm{th}}$	cm^2^-K/W	0	0.010	0.027	-
$C_{h}$	J/cm^3^-K	0	0	2.82	2.82 [52]
$d_{h}$	nm	0	0	157	-
$\bar{\epsilon }$	10^−3^ K^2^	39.2	2.23	3.01	

**Table A3:** Fitted parameter values for sample C.

Parameter.	Units	(i) $C_{h}d_{h},\;r_{th}=0$	(ii) $r_{th}\neq 0$	(iii) $C_{h}d_{h},r_{th}\neq 0$	Ref. Value
$\kappa_{\mathrm{BCB}}$	W/m-K	0.20 ± 0.03	0.19 ± 0.03	0.17 ± 0.03	0.18–0.29 [66,67,68,69]
$C_{\mathrm{BCB}}$	J/cm^3^-K	2.22 ± 0.05	1.85 ± 0.05	2.05 ± 0.05	2.19–2.29 [66,70]
$\kappa_{\mathrm{Si}}$	W/m-K	256	142	*5.03* × *10^4^*	145–156 [72,73]
$C_{\mathrm{Si}}$	J/cm^3^-K	1.87	1.82	1.91	1.66 [74]
$r_{\mathrm{th}}$	cm^2^-K/W	0	0.012	0.025	-
$C_{h}$	J/cm^3^-K	0	0	2.82	2.82 [52]
$d_{h}$	nm	0	0	157	-
$\bar{\epsilon }$	10^−3^ K^2^	143	1.30	11.7	

## Appendix C

The thermophysical properties of the bulk GaAs and InP substrates, which were measured to validate the experimental setup, are shown in Table A4 and Table A5 in this section, respectively. Figure A1 shows the measured temperature amplitudes and corresponding fit lines for both samples.

**Table A4:** Fitted parameter values for bulk GaAs.

Parameter	Units		Ref. Value
$\kappa_{\mathrm{GaAs}}$	W/m-K	49 ± 2	49–56 [19,52]
$C_{\mathrm{GaAs}}$	J/cm^3^-K	1.9 ± 0.1	1.8 [54]
$r_{\mathrm{th}}$	cm^2^-K/W	0.00011	-
$C_{h}$	J/cm^3^-K	2.82	2.82 [52]
$d_{h}$	nm	157	-
$\bar{\epsilon }$	10^−3^ K^2^	0.14	

**Table A5:** Fitted parameter values for bulk InP.

Parameter	Units		Ref. Value
$\kappa_{\mathrm{InP}}$	W/m-K	77 ± 2	70–80 [52,75]
$C_{\mathrm{InP}}$	J/cm^3^-K	1.5 ± 0.1	1.5 [76]
$r_{\mathrm{th}}$	cm^2^-K/W	0.00011	-
$C_{h}$	J/cm^3^-K	2.82	2.82 [52]
$d_{h}$	nm	157	-
$\bar{\epsilon }$	10^−3^ K^2^	0.15	

## Appendix D

**Table A6:** Abbreviations and symbols.

Abbreviations		Symbols	Symbols Units	
BCB	benzocyclobutene	ZT	thermoelectric figure of merit	1
MSE	mean squared error	S	Seebeck coefficient	V/K
NW	nanowire	σ	electrical conductivity	S/m
SA	self-assisted	T	absolute temperature	K
SAED	selective-area electron diffraction	κ	total thermal conductivity	W/m-K
SEM	scanning electron microscopy	$\kappa_{e}$	electronic thermal conductivity	W/m-K
TEM	transmission electron microscopy	$\kappa_{L}$	lattice thermal conductivity	W/m-K
TSL	twinning superlattice	ρ	electrical resistivity	Ω-m
VLS	vapor–liquid–solid	${\tilde{\theta }}_{2\omega ,\mathrm{rms}}$	measured temperature rise	K
WZ	wurtzite	$I_{1\omega ,\mathrm{rms}}$	measured source current	A
ZB	zincblende	dR/dT	heater line resistance coefficient	Ω/K
		$V_{3\omega ,\mathrm{rms},\mathrm{re}}$	measured in-phase 3ω voltage	V
		$V_{3\omega ,\mathrm{rms},\mathrm{im}}$	measured out-of-phase 3ω voltage	V
		ω	frequency	Hz
		${\tilde{T}}_{h}$	corrected heater temperature rise	K
		$\Delta \tilde{T}$	base heater temperature rise	K
		p	heat flux	W/m^2^
		$r_{\mathrm{th}}$	heater line thermal contact resistance	m^2^-K/W
		$C_{h}$	heater line heat capacity	J/m^3^-K
		$d_{h}$	heater line thickness	m
		P	peak electrical power	W
		l	heater line length	m
		$\kappa_{⊥,n}$	cross-plane thermal conductivity of layer *n*	W/m-K
		$C_{n}$	heat capacity of layer *n*	J/m^3^-K
		$d_{n}$	thickness of layer *n*	m
		$\psi_{n}$	thermal conductivity anisotropy ratio of layer *n*	1
		$A_{n}$	recursive coefficient Equation (5)	1
		$B_{n}$	coefficient Equation (5)	m^−1^
		b	heater line half-width	m
		λ	integration variable Equation (5)	m^−1^
		$\bar{\epsilon }$	mean squared error	K^2^
		$\vec{\chi }$	sample parameters vector	-
		$\kappa_{\mathrm{NW}-\mathrm{BCB}}$	measured NW-BCB thermal conductivity	W/m-K
		$\kappa_{\mathrm{NW}}$	measured NW array thermal conductivity	W/m-K
		$\kappa_{\mathrm{BCB}}$	measured BCB thermal conductivity	W/m-K
		$x_{A\left(B\right)}$	NW volume fraction in sample A(B)	1
		$D_{A\left(B\right)}$	NW diameter in sample A(B)	m
		$\kappa_{A\left(B\right)}$	NW array thermal conductivity in sample A(B)	W/m-K
