# Supplementary material for: Thermal Conductivity of GaAs Nanowire Arrays Measured by the 3ω Method
Source: Nanomaterials (Basel). 2022 Apr 10;12(8):1288. doi: 10.3390/nano12081288 (PMC9026786; doi:10.3390/nano12081288)
Supplement: Supplementary file 1 [file nanomaterials-12-01288-s001.zip › nanomaterials-1673058-supplementary.pdf]

Thermal conductivity reduction in GaAs nanowire arrays measured by the 3ω method: Supplementary Information

Ara Ghukasyan, Pedro Oliveira, Nebile Goktas, and Ray LaPierre

Department of Engineering Physics, McMaster University
1280 Main Street W., Hamilton, ON, Canada, L8S 4L7

I. Heater Line Resistance Coefficients

The measured $R(T)$ data points, from which the resistance coefficients are determined, are shown in Figures S1-S3 for samples A, B, and C, respectively. In each case, the dashed line represents the best-fit and corresponding slope, whereas the blue and red lines indicate the minimum and maximum slope, respectively, within limits of the measurement uncertainty. Owing to the precision of the resistance measurements, a maximum error of $\pm 1.6\%$ was observed for the slope ($\mathrm{d}R/\mathrm{d}T$) across all samples; hence we determine the resistance coefficients to within 2% error.

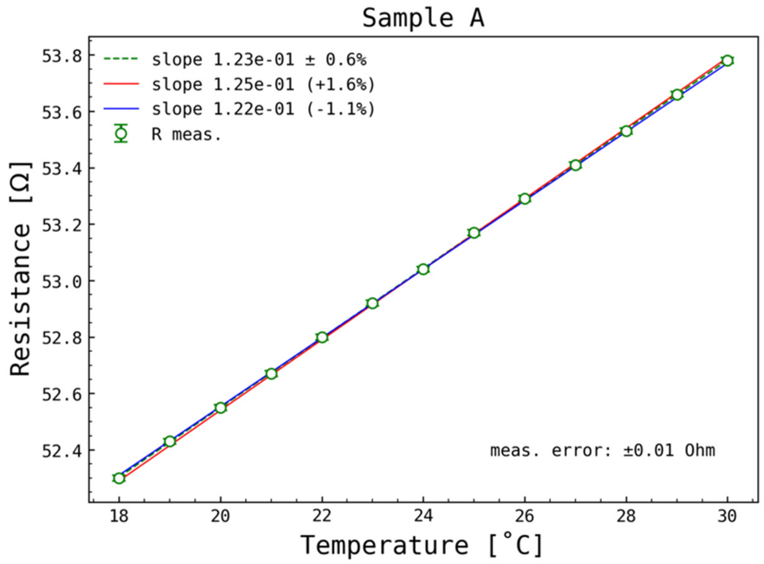

Figure S1: Temperature dependence of the line resistance for sample A.

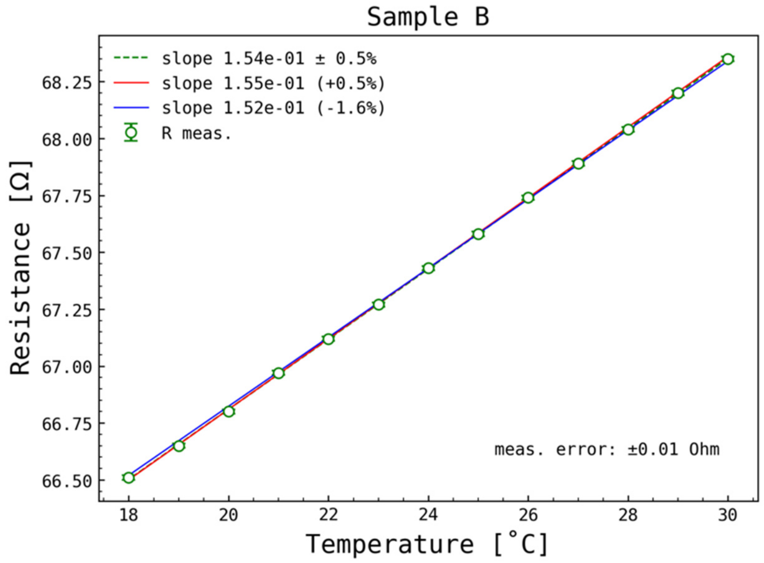

Figure S2: Temperature dependence of the line resistance for sample B.

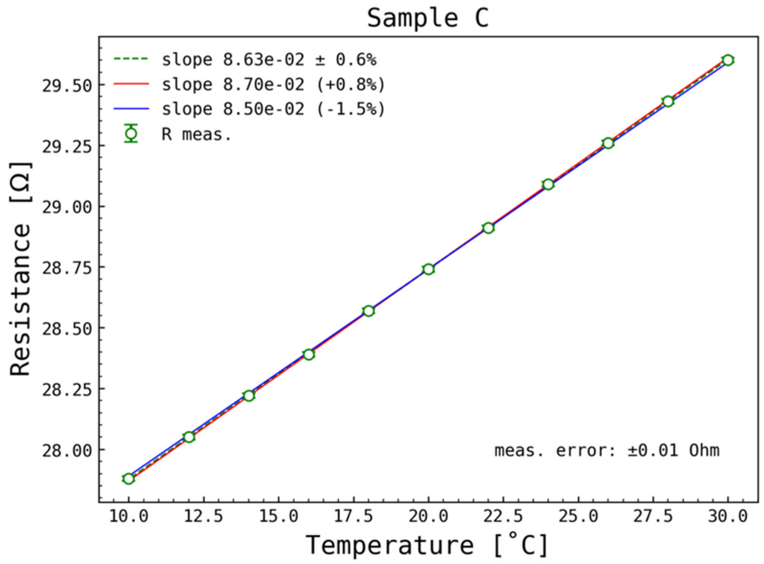

Figure S3: Temperature dependence of the line resistance for sample C.

II. Model Sensitivity

To illustrate the sensitivity of the mean-squared-error objective function,

$$\bar{\epsilon}(\vec{x}) = \frac{1}{M}\sum_{k=1}^{M}\left\|\tilde{\theta}_{2\omega_k} - \tilde{T}_h(\omega_k, \vec{\chi})\right\|^2 \qquad \text{(S1)}$$

to individual parameter values, χ_i, we varied the ith component of best fit vector $\vec{\chi}^*$ by $\pm 50\%$ and recorded the resulting change in $\bar{\epsilon}$. Parameters exhibiting high sensitivity are marked by a steep descent into a pronounced minimum for $\bar{\epsilon}$ at χ_i^* (i.e., near 0% perturbation). Conversely, the variation yields much smaller changes for low-sensitivity parameters, namely the heat capacities of layers (2) and (3) in samples A and B. This is evident in Figure S4, where $\vec{\chi}^*(B)$ was varied by perturbing the layer properties of sample B. Figure S4 also demonstrates that the properties of the uppermost layer have the greatest influence on $\bar{\epsilon}$. Thus, the accuracy of the measurement increases with proximity of the target layer to the heater line.

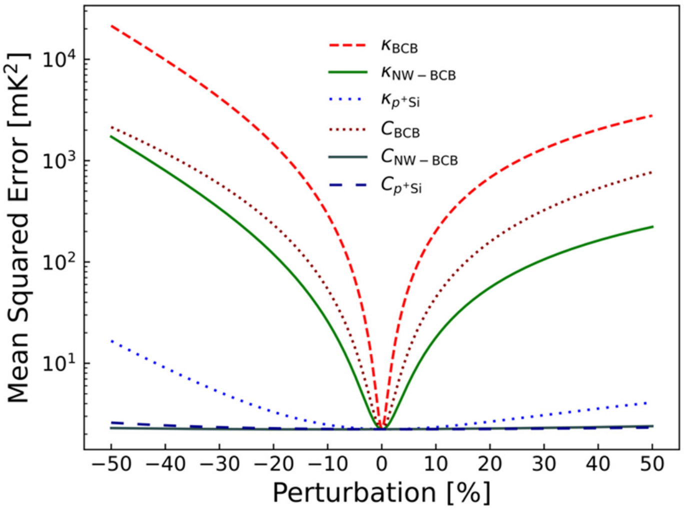

Figure S4: Sensitivity of the objective function versus perturbations about the best fit values. The upper three curves indicate a strong sensitivity to the thermal conductivities of the BCB and NW-BCB layers, as well as the volumetric heat capacity of the BCB. A weaker sensitivity to the substrate (p^+-Si) thermal conductivity is also seen.

While the theoretical heat model surmised by Eqs. (4) to (7) in the main text can in principle include an arbitrary number of layers, the effective penetration depth [1],

$$q^{-1} = \sqrt{\frac{\kappa}{2\omega C}} \tag{S2}$$

must be taken into consideration vis a vis the total thickness of the sample and constituent layers. Figure S5 shows the approximate penetration depth through BCB and the NW-BCB composite, calculated from Eq. (S2), using our results in Tables A1-A3 in the main text. In both samples A and B, the BCB and composite layers had a combined height of about 6 μm, whereas sample C had only the 3 μm of BCB. Accordingly, the plot of the penetration depth, Figure S5, indicates complete thermal probing in all three measurement devices, over nearly all the measured frequencies.

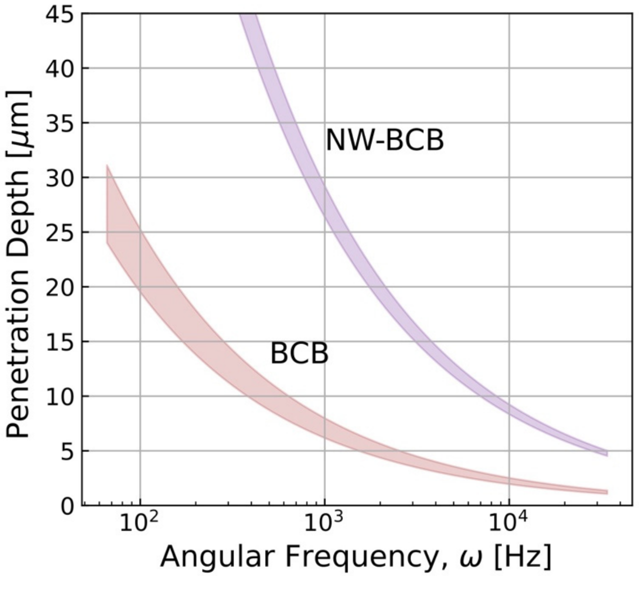

Figure S5: Thermal penetration depth (q^{-1}) in μm, calculated using Eq. (S2), using the range of values for BCB and the NW-BCB composite from Tables A1-A3 in Appendix B of the main text.

References

1. Borca-Tasciuc, T.; Kumar, A.R.; Chen, G. Data Reduction in 3ω Method for Thin-Film Thermal Conductivity Determination. *Rev. Sci. Instrum.* **2001**, *72*, 2139–2147, doi:10.1063/1.1353189.
